# Special Hospitals

**Published:** 1891-12-19

**Authors:** 


### SPECIAL HOSPITALS.

### CONSUMPTION.

**Brompton Hospital for Consumption and Diseases of the Chest,** S.W.—This hospital is well known as one where the patients are well cared for, and every effort is made to brighten their clouded lives. The maintenance of the new extension building entails an extra expenditure of several thousand pounds a-year, and funds are urgently needed to meet this outlay. Secretary, Mr. H. Dobbin; Matron, Miss F. Abbott.

**City of London Hospital for Diseases of the Chest,** Victoria Park, E.—The average expenditure is about £10,000, towards which only £370 of the income assured. Altogether about 1,000 in-patients and 15,000 out-patients are treated yearly. Secretary, Mr. T. Storrar-Smith; Matron, Miss H. G. Hetherington.

**North London Hospital for Consumption,** Mount Vernon, Hampstead, N.W.—This institution was thoroughly reorganised and remodelled some years ago, and has now become one of the most useful of its class. Though not so large as the above, yet 400 in, and 3,000 out-patients are admitted each year. Secretary, Mr. Lionel F. Hill, M.A.; Matron, Miss Blaxland.

**Royal Hospital for Diseases of the Chest,** City Road, E.C.—Five hundred and twenty-three in-patients and 8,662 out-patients were relieved in 1890. Seven-eighths of the patients are of the hard-working and wage-earning class. Two out of the four wards in the wing opened in 1886 are at the present time closed for want of funds. Secretary, Mr. John Harrold; Matron, Miss M. L. Smith.

### EPILEPSY AND PARALYSIS.

**Hospital for Epilepsy and Paralysis,** Portman Terrace, Regent's Park, N.W.—At this institution, in order to promote providence and to minimise abuse, patients are encouraged to pay what they can afford. The payments from these patients does not, however, nearly cover the cost of the treatment, while those unable to pay receive free relief. The hospital being unendowed, there necessarily remains a large sum to be collected each year, and the Committee, therefore, are appealing for at least £1,000 in new annual subscriptions. Secretary, Mr. Howgrave Graham; Matron, Miss Ridley.

**National Hospital for Paralysed and Epileptic,** Queen Square, W.C.—This hospital relieves a class of sufferers terribly afflicted, large in numbers, and unfitted for general hospitals for whom little provision is made in any other institution. It provides 170 beds and a spacious out-patients' department, while a surgical wing is also in course of erection. It is manifest how insufficient less than 200 beds must be to meet the large demands made upon this class of hospital, and as a matter of fact, although the accommodation is taxed to the utmost, there is always a long list of patients, medically selected, who are waiting to come in. One great need is an extension of the annual subscription list which, relatively to the expenditure (£1,800 to £13,000), is very inadequate. To increase the reliable income is the present chief need of the charity, and then to still further extend its accommodation. Secretary, Mr. B. Burford Rawlings; Matron, Miss L. C. East.

### CHILDREN.

**East London Hospital for Children,** Shadwell, E.—An excellent institution, the original home of which in Ratcliff Highway was graphically described by Charles Dickens, whose memorable account will live for generations. Unfortunately, the children admitted are in as dire straits now as then, and funds are sadly needed. Secretary, Mr. S. Whitford; Matron, Mrs. Fisher; Lady Superintendent, Miss F. A. Davies.

**Evelina,** Southwark Bridge Road, S.E.—The only hospital in South London exclusively for children, and situated in a district where nearly all the resident inhabitants may be numbered among the poor. Any poor sick or suffering child is admitted if there is room. It is open to visitors every day, from two to four. Contributions, which could not be better bestowed, may be sent to the Secretary, Mr. T. S. Chapman; Lady Superintendent, Miss Alice Cross.

**Hospital for Sick Children,** Great Ormond Street, W.C.—The 127 beds which the hospital at present contains being found totally inadequate, a new wing is being erected with 93 additional cots. This is now nearly completed, but before it can be opened a further £8,000 must be collected to pay for furniture and fittings. Besides this, £1,400 is still required to meet the expenses for the last quarter of this year. Seeing that this is the largest children's hospital in Great Britain, that it is excellently managed, and that it has treated over 1,400 in-patients and 20,500 out-patients during 1890, we do not think that this year's appeal to the charitable public for funds to enable them to carry on the great work will be made in vain. Secretary, Mr. Adrian Hope; Lady Superintendent, Miss K. Hendle Close.

**North-Eastern Hospital for Children,** Hackney Road, E.C.—This institution has prospered greatly since Mr. Alfred Nixon became Secretary some years ago, and its present condition shows what can be done by an energetic management. The average number of out-patients during the last ten years is 13,500, and last year 702 in-patients were treated. Patients are admitted from all parts of London and the United Kingdom. Secretary, Mr. Alfred Nixon; Matron, Miss E. W. Curno.

**Paddington Green Children's Hospital, W.**—Though this hospital is in the fortunate position of being free from debt on the current expenditure, yet it this year makes a special appeal for the purpose of raising funds to enable the committee to add a wing in order to provide, as far as possible, for the number of cases which they are now obliged to reject for want of room. The cost will probably amount to £4,000 or £5,000, but the committee have determined not to commence building until a considerable proportion of this has been raised. Secretary, Mr. W. H. Pearce; Matron, Miss Anderson.

**Victoria Hospital for Children,** Queen's Road, Chelsea.—Established at Chelsea in 1866 for the relief of the sick and suffering children of the poor, this unendowed hospital seeks further subscriptions to help it to carry on its work. The expenditure, which is only 19s. per patient, yet reaches £5,000 to £6,000 a-year, and this has to be raised entirely by voluntary subscriptions. Secretary, Commander W. C. Blount, R.N.; Matron, Miss Cooper.

### LYING-IN.

**Queen Charlotte's Lying-in Hospital,** Marylebone Road, N.W.—This hospital appeals to all wives and mothers, as here a home is given to poor women at the time they most need care and skilful treatment. Secretary, Mr. G. Owen Ryan; Matron, Mrs. Phillips.

**Royal Maternity Charity,** 31, Finsbury Square, E.C.—One of the oldest and largest lying-in charities in Great Britain, attending some 4,000 poor women annually at their own homes, this charity appeals to all. Under the system of home attendance the limit to the charity's work is set only by the funds it has to deal with. Donations and legacies are greatly required. Secretary, Mr. T. W. Long.

### WOMEN.

**Chelsea Hospital for Women,** Fulham Road, S.W.—This hospital has 60 beds, and treats respectable poor women and ladies in reduced circumstances who are victims of ills peculiar to their sex. Contributing in-patients occupy separate wards, paying 10s. 6d. to 42s. a-week, according to their means. The hospital is entirely without endowment or reserve funds, and earnestly desires more annual subscriptions from women who have pity on their sex. Secretary, Mr. A. C. Davis; Matron, Miss Wade.

**Grosvenor Hospital for Women and Children,** Vincent Square, Westminster, S.W.—The Grosvenor Hospital is situated in a densely-populated district of Westminster, and the bulk of the out-patients is drawn from the poorer classes living in the neighbourhood. In-patients, however, are received from all parts of the country, and both residents and non-residents of London may well give the institution a generous support. The hospital has been lately enlarged to meet the increased demands for its benefits, and the current annual expenditure is consequently increased. Secretary, Hon. F. C. Howard; Matron, Miss K. Hughes.

**New Hospital for Women,** 144, Euston Road, N.W.—The principal feature of the hospital is that it enables poor women to consult qualified physicians of their own sex; and this boon has been so highly appreciated that the work begun in 1872, with ten beds, has increased to such an extent that last year a new hospital, with 42 beds, was opened for the reception of in-patients. The beds have been continuously occupied ever since, and numbers are waiting for admission.—Secretary, Miss M. M. Bagster.

**Samaritan Free Hospital for Women and Children,** Marylebone Road, N.W.—Some £7,000 per annum is required to maintain this hospital, of which only £1,750 can be relied upon in annual subscriptions, for the rest it has to depend entirely on donations—a most uncertain source of income—bequests and awards from the Hospital Sunday Fund and Hospital Saturday Fund, &c. The Committee would therefore like to see a great addition to the list of annual and life subscriptions. Secretary, Mr. G. Scudamore; Matron, Miss Butler.

**The Hospital for Women,** Soho Square, W.—This institution attains its jubilee next year, and to mark this the Committee hope to receive sufficient funds to enable them not only to maintain all the 66 beds in constant use, as at present, but also to provide much needed additional accommodation for the out-patient department and the nursing staff by rebuilding some of the present quarters for both. During the past three years a large proportion of the income of the hospital has been derived from legacies; but as this source is always a very precarious one, the Committee very earnestly appeal for additional annual subscriptions in order that the reliable income of the charity may be raised to an amount more commensurate with its requirements. Secretary, Mr. David Cannon; Matron, Miss Squier.

**The Royal Hospital for Children and Women,** Waterloo Bridge Road, S.E.—This hospital stands in the midst of the densest poverty—indeed, with the single exception of St. George's-in-the-East, the population is more crowded than that of any other portion of London, being 216 persons to the acre. This year the demand on the beds has been exceptionally high, yet the income for the year is insufficient to enable the hospital to continue to keep open all the wards unless further help is obtained. A sum of £1,000 is wanted, and that immediately, if this valuable institution is adequately to perform its good work. Secretary, Mr. R. G. Kestin; Matron, Miss Mary Mocatta.

### MISCELLANEOUS.

**Cancer Hospital,** Fulham Road, Brompton, S.W.—This free hospital, founded in 1851, shelters over six hundred sufferers, not only from this most painful disease, but also from many species of tumours which, if neglected, might prove fatal, but, if dealt with in time, are curable; it also benefits a large number of out-patients. The annual expenditure is about £3,000 in excess of the reliable income, to meet which new annual subscriptions and donations are urgently needed. Secretary, Mr. W. H. Hughes; Matron, Miss A. Rogers.

**Central London Throat and Ear Hospital,** Gray's Inn Road, W.C.—The hospital was established eighteen years ago for the treatment of poor patients suffering from diseases of the throat, nose, and ear. It is situated in a very poor and populous district, while its contiguity to the various railway termini at King's Cross renders it easily accessible for patients from all parts of London, the suburbs, and the country. The hospital has no endowment whatever, and is dependent entirely upon voluntary contributions. Secretary, Mr. R. Kershaw; Matron, Miss M. Danter.

**Hospital for Diseases of the Throat,** Golden Square, W.—Owing to the very large proportion of children amongst the patients treated here, the Committee have, during the past year, had their Board-room converted into a children's ward. The Committee have no funds to meet the additional expense thus entailed, and they earnestly hope that some portion of the charitable gifts of the season will be devoted to this object. They have, moreover, urgent need of increased out-patients accommodation, and they are endeavouring to collect a special fund for this purpose; they have acquired premises adjacent to the hospital, but will not be able to convert them until they have obtained a fund of at least £2,000. Secretary, Mr. W. Thornton Sharp; Matron, Miss M. B. Mackey.

**Lock Hospital and Asylum,** Harrow Road, W.—Rescue work, as well as the cure of the patients who present themselves for admission, is the aim of this institution, and if it were more generally known what good work is being done by its means, we feel sure that it would meet with the support it deserves. The result, that considerably more than one-fourth of all those that pass through this hospital are reclaimed from their evil lives, points to a great work, which should be far more encouraged. A debt of £4,600 has still to be cleared off, and at least £1,500 is required in fresh annual subscriptions to meet the regular requirements of the institution. Matron of hospital, Miss Kydd; Matron of asylum, Miss J. McNiven.

**Royal London Ophthalmic Hospital**, Moorfields, E.C.—An urgent appeal is made by the Board of Management for aid in the erection of a new hospital on ground recently leased to them by the City of London, and in the re-arrangement of the old hospital adjoining, to meet the medical and sanitary requirements of the day. The lowest estimated cost of the new building and alterations alone is £35,000, exclusive of cost of refurnishing, and additional annual expenditure consequent on the increase in size. All patients are admitted free, and without letters of recommendation. Secretary, Mr. Robert J. Newstead; Matron, Miss Nichol.

**Royal South London Ophthalmic Hospital**, St. George's Circus, S.E.—The work of this institution has been hitherto carried on in a building consisting of two dwelling-houses, but a new hospital has now been erected, and will be occupied in the early part of 1892. It will contain forty beds, and though funds are in hand for completing it, it will have to entirely depend for maintenance upon charitable contributions.—Secretary, Mr. Chas. Comyn.

